# Mechanisms involved in cefiderocol resistance in French *Pseudomonas aeruginosa* clinical strains

**DOI:** 10.1128/aac.00520-26

**Published:** 2026-07-10

**Authors:** Emma Gauthier, Matthieu Pisani, Maxime Bour, Mélanie Grosjean, Patrick Plésiat, Shahriar Safari, Ruben C. Hartkoorn, Léa Souro, Emma Prétot, Katy Jeannot

**Affiliations:** 1 Laboratoire de Bactériologie, CNRS, Chrono-environnement (UMR 6249), Université Marie et Louis Pasteur, CHU Besançon Franche-Comté 27000, Besançon, France; 2 RD-Biotech Company, Besançon, France; 3 Université de Lille, CNRS, Inserm, CHU Lille, Institut Pasteur Lille, U1019-UMR 9017, Center for Infection and Immunity of Lille (CIIL) 27023, Lille, France; Entasis, Big Bay, Michigan, USA

**Keywords:** cefiderocol, *Pseudomonas aeruginosa*, antimicrobial resistance

## Abstract

Cefiderocol exhibits excellent *in vitro* activity against *Pseudomonas aeruginosa*; however, resistance can emerge. We investigated the molecular mechanisms underlying cefiderocol resistance (minimal inhibitory concentration [MIC] >2 mg/L) in 103 clinical strains collected from 61 hospitals (2021–2024). MICs ranged from 4 to >128 mg/L, with 39.8% of strains showing MICs > 8 mg/L. Although 37.8% were classified as difficult-to-treat resistant (DTR), acquired β-lactamases were detected in 72.8% of strains, including carbapenemases (39.8%), mainly NDM-1 (29.1%), and extended-spectrum β-lactamases (ESBLs) (38.8%). Cloning of 11 β-lactamases into pUCP24, including the acquired cephalosporinase PAC-1 and ESBLs (VEB-1 and VEB-9), resulted in marked increases in cefiderocol MICs (up to 128-fold). Introduction of six mutations in the PDC enzyme into a PAO1Δ*bla*_PDC-1_ background increased MICs up to 4 mg/L and conferred cross-resistance to ceftolozane/tazobactam, notably T70I, F121L, G157D, and E219K. Alterations in siderophore transporters or regulators were identified in 38.8% of strains, most frequently a PirR frameshift (R132*fs*), consistent with PirR inactivation, which was confirmed in the PAO1 strain to contribute to cefiderocol resistance. Overall, cefiderocol resistance in clinical strains is multifactorial, mainly involving acquired β-lactamases (ESBLs and carbapenemases) and impaired siderophore uptake (PiuA/PiuD, PirA, and PiuC), leading to high-level resistance (>8 mg/L). The polyclonal distribution and diversity of mechanisms highlight the need for routine susceptibility testing and surveillance. Detection of NDM producers is critical, as cefiderocol should be used with caution in this context.

## INTRODUCTION

*Pseudomonas aeruginosa* is notoriously known as the most common cause of healthcare-associated pneumonia in intensive care units (20.1% in Europe) (1). While most isolates (67.6%) remain susceptible to first-line antibiotics such as ceftazidime, piperacillin/tazobactam, and meropenem, 11.3% of invasive European isolates are multidrug-resistant (MDR), extensively drug-resistant (XDR), or difficult-to-treat resistance (DTR), limiting therapeutic options to a few antibiotics including cefiderocol (2). This first cephalosporin-siderophore is currently a therapeutic option in adults to combat *P. aeruginosa* infections associated with isolates resistant to carbapenems, ceftazidime/avibactam, ceftolozane/tazobactam, and imipenem/cilastatin/relebactam (3). While this cephalosporin-siderophore generally has excellent *in vitro* activity against such *P. aeruginosa* clinical isolates, several studies have reported several intrinsic and acquired mechanisms of resistance to cefiderocol (4–6). In relation to its active entry into the periplasmic space, loss-of-function mutations in the TonB-dependent siderophore transporters PiuA or PiuD (depending on the genetic background of the strain) have been reported to lead to 16-fold and 32-fold increases in cefiderocol MIC in both the PAO1 reference strain and the clinical strain after a ceftazidime/avibactam exposure (7–10). Recently, mutations of the cognate TonB-receptor FptA (the receptor for pyochelin, the second endogenous *P. aeruginosa* siderophore) were associated with a 2-fold to 4-fold decrease in cefiderocol susceptibility in the PA14 reference strain (11). In addition, while cefiderocol is active against *P. aeruginosa* AmpC-overproducing strains, certain mutations in AmpC (such as T96I, F121L + G222S, G183D, G214R, E219K, E247K, and L320P), when expressed recombinantly in PAO1 or in PAO1Δ*ampC*Δ*oprD*, have been shown to impact cefiderocol in *vitro* activity (12, 13). Of these PDC variants, the E247K mutation has also been reported in two clinical cefiderocol-resistant strains of *P. aeruginosa* (with MIC values of 8 and 32 mg/L) according to EUCAST breakpoints (MIC >2 mg/L) following exposure to ceftolozane/tazobactam (14). Additionally, a four-amino acid deletion (TPMA) at positions 316–319 of PDC was identified in a clinical strain of *P. aeruginosa* (from a patient previously treated with cefiderocol)*,* which led to a significant increase in the cefiderocol MIC (from 0.12 to 2 mg/L) (15). Beyond mutation-driven intrinsic resistance mechanisms, various types of ß-lactamases, when expressed recombinantly, have been shown to mediate cefiderocol resistance, including carbapenemases (PER-7, NDM-1, and SPM-1), extended-spectrum ß-lactamases (PER-1 and -6; SHV-2a and -12; and BEL-1 and -2), or oxacillinases derived from OXA-2 (OXA-2, -15, -161, -226, -539, -681, -737, and -935), OXA-10 (OXA-14, -19, -21, -794, -795, -824, and -836), and OXA-46 (OXA-46 and -779) (13, 16, 17). Despite the various potential mechanisms of cefiderocol resistance identified, there have been limited reports on the diversity of such mechanisms in cefiderocol-resistant *P. aeruginosa* clinical strains (4, 18). The aim of this study was to map the genetic mechanisms contributing to cefiderocol resistance (MIC >2 mg/L) in a French collection of 103 cefiderocol-resistant *P. aeruginosa* clinical strains.

## RESULTS

From the collection of the French National Reference Center (FNRC) for antibiotic resistance *in P. aeruginosa*, 103 clinical strains (covering 4 years) were considered resistant to cefiderocol according to its clinical breakpoints defined by EUCAST 2025 (MIC >2 mg/L). These strains were originally isolated from the respiratory tract (29.1%), blood (22.3%), urine (18.4%), rectal swabs (9.7%), bone (8.7%), skin (6.8%), and other sites (5.0%) (Table S1). The cefiderocol susceptibility of these strains ranged from an MIC of 4 (*n* = 35, 34.0%) to ≥128 mg/L (*n* = 8, 7.8%), with 41/103 strains (39.8%) having MIC values higher than 8 mg/L, corresponding to the cefiderocol resistance according to the CLSI breakpoints 2025 (19) (Table 1). The cefiderocol-resistant strains were highly resistant to first-line antibiotics (piperacillin/tazobactam [90.3%], ceftazidime [95.1%], and cefepime [96.1%]) and carbapenems (imipenem [81.5%] and meropenem [71.8%]), as well as to combinations of ß-lactam and ß-lactamase inhibitors (ceftolozane/tazobactam [90.3%] and ceftazidime/avibactam [89.3%]) (Table 1; Table S1). Aztreonam (66.0%) and imipenem/relebactam (68.9%) were the only ß-lactams for which resistance rates were found to be below 70%. When integrating fluoroquinolone susceptibility into the analysis, a total of 39 cefiderocol-resistant strains (37.8%) could be classified as DTR (20). These DTR strains were also resistant to ceftolozane/tazobactam (35/39 DTR strains), ceftazidime/avibactam (30/39 DTR strains), imipenem/relebactam (36/39 DTR strains), and amikacin (33/39 DTR strains), although most remained sensitive to colistin (resistance found in 2/39 DTR strains).

**TABLE 1:** Antibiotic MIC distribution of 103 cefiderocol-resistant clinical strains of *P. aeruginosa*

Antibiotics	MIC (mg/L)^*a*^										Percentage of resistant (*N*)
	≤0.25	0.5	1	2	4	8	16	32	64	≥ 128	
Piperacillin/tazobactam^*d*^					2^*b*^	4	4	11	7	75	90.3 (93)
Ceftazidime				1	3	1	3	1	4	90	95.1 (98)
Cefepime				2		2	4	12	14	69	96.1 (99)
Ceftazidime/avibactam^*e*^			1	1	7	2	10	8	12	62	89.3 (92)
Ceftolozane/tazobactam^*f*^			2	4	4	7	1	4	7	74	90.3 (93)
Cefiderocol					35	27	25	3	3	10	100 (103)
Aztreonam				3^*b*^	9	9	14	10	17	41	66.0 (68)
Imipenem		1^*b*^	5	8	5	12	18	13	2	39	81.5 (84)
Imipenem/relebactam^*g*^		12^*b*^	5	15	14	13	6	0	1	37	68.9 (71)
Meropenem		3^*b*^	2	4	3	17	17	15	3	39	71.8 (74)
Amikacin				5	9	9	6	14	2	58	71.8 (74)
Ciprofloxacin	12	7	2	1	7	74^*c*^					81.5 (84)
Colistin		6^*b*^	60	31	1	2	1	2^*c*^			4.8 (5)

^*a*^ The shaded area highlights the strains categorized drug-resistant according to the EUCAST 2025 (Breakpoints, 2025 #3057).

^*b*^ MIC less than or equal to the indicated value.

^*c*^ MIC higher than or equal to the indicated value.

^*d*^ Fixed concentration of tazobactam at 4 mg/L.

^*e*^ Fixed concentration of avibactam at 4 mg/L.

^*f*^ Fixed concentration of tazobactam at 4 mg/L.

^*g*^ Fixed concentration of relebactam at 4 mg/L.

**Fig 1:**
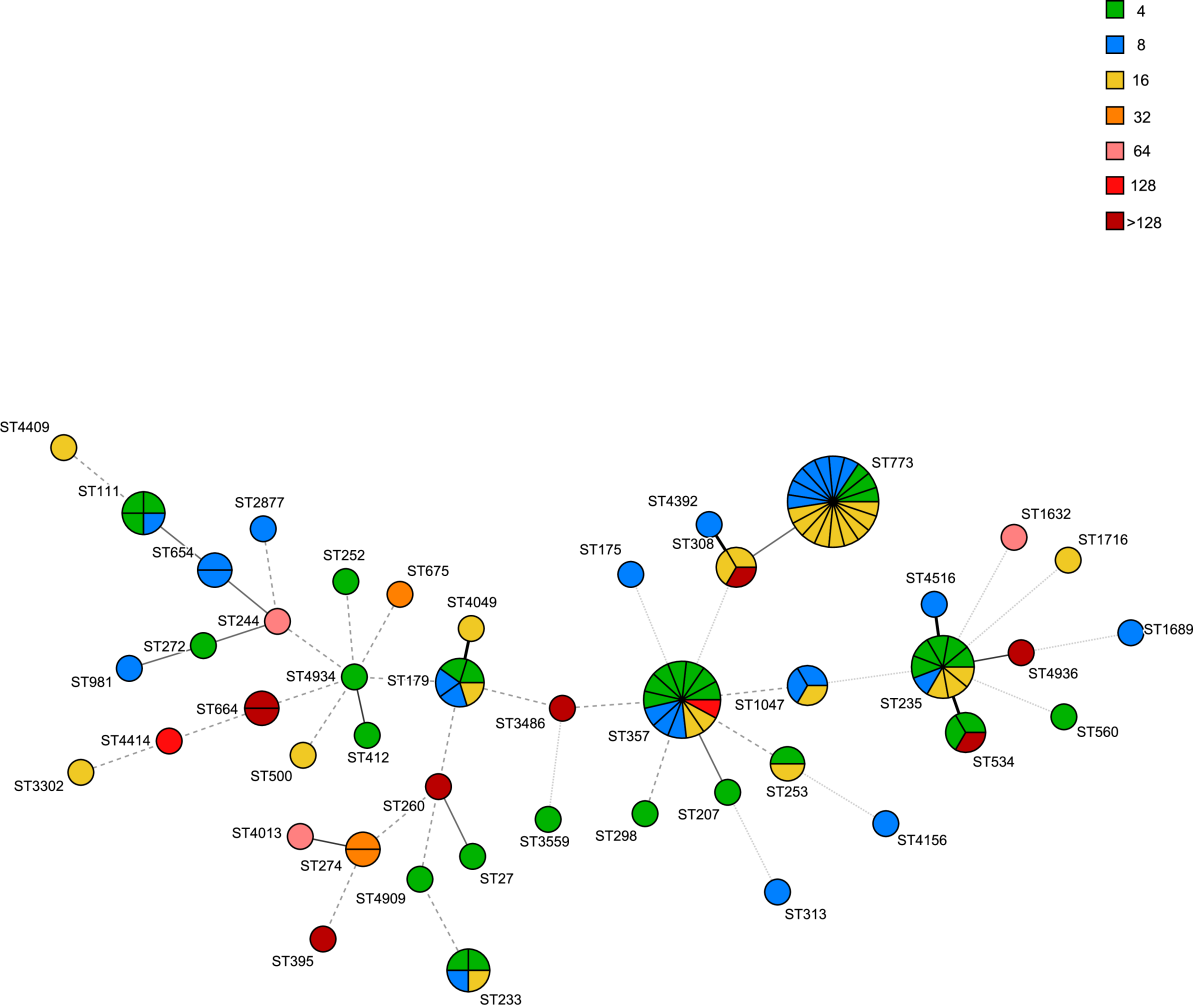
Sequence type (ST) distribution of 103 cefiderocol-resistant strains. The MIC values of cefiderocol for the studied strains are represented as follows: green for 4 μg/mL, blue for 8 μg/mL, yellow for 16 μg/mL, orange for 32 μg/mL, pink for 64 μg/mL, and red for ≥ 128 µg/mL. The lines connecting the STs represent the number of allelic differences between them. A solid line, a stippled line, and a very fine dotted line indicate 2, 3, and 4 allelic differences between STs, respectively. The minimum spanning tree was drawn on BioNumerics 7.1 and modified on Inkscape 1.4.2 software.

When examining the sequence type (ST) of these strains, a total of 45 STs were identified, with only 6 STs represented by more than three strains (specifically, ST111 [*n* = 4], ST179 [*n* = 5], ST233 [*n* = 4], ST235 [*n* = 8], ST357 [*n* = 13], and ST773 [*n* = 19]) (Fig. 1). With the exception of ST179 and ST773, the STs associated with cefiderocol-resistant strains were previously classified among the top 10 high-risk lineages found to accumulate resistance determinants (21). Overall, the cefiderocol-resistant strains were polyclonal, with cefiderocol susceptibility not correlating with ST, which implies that the probability of cefiderocol resistance cannot be predicted in routine clinical laboratories by ST determination.

### Transferable resistance mechanisms in cefiderocol-resistant clinical strains

Whole genome sequencing data were used to map the acquisition of transferable β-lactamases in the 103 cefiderocol-resistant strains. Of these strains, a total of 77/103 (74.7%) were found to have acquired at least one transferable ß-lactamase, including carbapenemases (41/103 strains, 39.8%), ESBL (40/103 strains, 38.8%), penicillinases (23/103 strains, 22.3%), and cephalosporinase PAC-1 (4/103, 3.8%) (Table 2; Table S2). Twenty-nine cefiderocol-resistant strains (28.1%) were identified to carry multiple ß-lactamases, of which 12 strains carried three such degrading enzymes (9.7%). Among these, 9/29 strains carried a carbapenemase and an ESBL, and 3/29 strains carried two distinct carbapenemases (AFM-5/IMP-26, VIM-4/OXA-641, and VIM-4/NDM-1). Of the 41 carbapenemase-producing strains, metallo-β-lactamases (MBL) were the most commonly identified, specifically NDM-1 (*n* = 30), IMP-1 (*n* = 2), IMP-13 (*n* = 2), IMP-26 (*n* = 1), VIM-1 (*n* = 1), VIM-2 (*n* = 3), VIM-4 (*n* = 2), AFM-2 (*n* = 1), and DIM-1 (*n* = 1), with only one strain identified to carry an oxacillinase (OXA-641) with carbapenemase activity.

**TABLE 2:** Resistance mechanisms identified in cefiderocol-resistant (MIC > 2 mg/L) *P. aeruginosa* clinical strains (*N* = 103)^*a*^

ST type^*b*^ (number of strains)	PDC^*c*^ type	MIC of FDC (mg/L)	Acquired β–lactamases (number of strains)	PBP-3	Proteins involved in siderophore transport and/or regulation										
					PiuA^*d*^	PiuD^*d*^	PiuC	PirA	PirS	PirR	FptA	FpvB	FiuA	FecA	PfeA
ST27 (*n* = 1)	**PDC-515**	4	–	–	–		–	–	–	–	–	–	–	–	–
ST111 (*n* = 4)	PDC-3	8	OXA-19, CARB-2	–		–	–	–	–	–	L439R	–	–	–	–
		4	IMP-13, OXA-4, CARB-2 (*n* = 2)	–		–	–	–	–	–	–	–	–	–	–
		4	OXA-35, CARB-2, OXA-9	–		–	–	–	–	*–*	–	–	–	–	–
ST175 (*n* = 1)	PDC–1	8	VIM-4, OXA-641, CARB-2	–		–	–	–	–	–	–	–	–	–	–
ST179 (*n* = 5)	PDC-8	16	PER-1	R504C	-		–	–	–	–	T68I	–	–	–	–
		8	PER-1, OXA-4 (*n* = 2)	R504C	–		–	–	–	–	–	–	–	–	–
		4	PER-1, OXA-4	R504C	–		–	–	–	–	–	–	–	–	–
		4	PER-1	R504C	–		–	–	–	–	T68I	–	–	–	–
ST207 (*n* = 1)	PDC-30	4	–	–	–		–	–	–	–	–	–	–	–	S2P
ST233 (*n* = 4)	PDC-3	16	NDM-1	R504C		–	–	–	–	–	–	–	–	–	–
		8	OXA-19	–		–	–	–	–	–	–	–	–	–	–
		4	OXA-19	–		–	–	–	–	–	–	–	–	–	–
		4	VIM-2, PER-1, OXA-4	–		–	–	–	–	–	–	–	–	–	–
ST235 (*n* = 9)	PDC-35	16	NDM-1	–		–	–	–	–	–	–	–	–	–	–
		16	IMP-26, ACA-7, AFM-5	–		*–*	*–*	–	–	–	–	–	–	–	–
		16	OXA-19	G531D		*–*	*–*	–	–	–	–	–	Q305*fs*	–	–
		8	GES-27	–		–	–	–	–	–	–	–	–	–	–
		4	OXA-19	–		–	–	–	–	–	–	–	–	–	–
		4	VIM-2, GES-1, OXA-10	–		–	–	–	–	–	–	–	–	–	–
		4	SHV-2a	–		–	–	–	–	*-*	–	–	–	–	–
		4	GES-1 (*n* = 2)	–		–	–	–	–	*–*	–	–	–	–	–
ST244 (*n* = 1)	PDC-1	64	OXA-1170	–	K42*fs*		–	–	–	–	–	–	M270I	–	–
ST252 (*n* = 1)	**PDC-334**	4	-	–	–		–	–	–	–	–	–	–	–	–
ST253 (*n* = 2)	PDC-34	16	OXA-35	–	–		–	–	–	R132*fs*	–	–	–	–	–
		4	–	–	–		–	–	–	–	–	–	–	–	–
ST260 (*n* = 1)	PDC-117	>128	OXA-17, OXA-142	–		–	–	–	–	R132*fs*	–	–	–	–	–
ST272 (*n* = 1)	PDC-5	4	VEB-1	–	-		–	–	–	–	–	–	–	–	–
ST274 (*n* = 2)	PDC-24	32	NDM-1	–		–	–	G363S,	–	–	–	–	–	H373R	–
	**PDC-230**	32	–	T77A N427S R504C		–	–	G363S, T424*fs*	P114S	R132*fs*	–	–	–	–	P80*fs*
ST298 (*n* = 1)	PDC-16	4	PER-1	–		–	–	–	–	–	–	–	–	–	T641I
ST308 (*n* = 3)	PDC-19	>128	NDM-1, PAC-1, OXA-10	–	–		–	–	–	*–*	–	–	–	–	–
		16	NDM-1	–	–		–	–	–	–	–	–	–	–	–
		16	NDM-1, PME-1	–	–		*–*	–	–	–	–	–	–	–	–
ST313 (*n* = 1)	PDC-37	8	–	–	T176A		–	–	–	R132*fs*	–	–	–	–	–
ST357 (*n* = 13)	PDC-11	128	GES-9, LCR-1	–		–	–	–	–	–	–	–	–	–	–
		16	NDM-1, LCR-1	–		–	–	–	–	–	–	–	–	–	–
		16	VEB-20, OXA-10	–		*–*	*–*	–	–	–	–	–	–	–	–
		8	NDM-1, VEB-9, OXA-10	–		–	–	–	–	–	–	–	–	–	–
		4	VIM-2, VEB-9, OXA-520	–		–	–	–	–	–	–	–	–	–	–
		4	VEB-9 (*n* = 4)	–		–	–	–	–	–	–	–	–	–	–
		4	GES-9, NPS-1	–		–	–	–	–	–	–	–	–	–	–
		4	VEB-9, OXA-677	–		–	–	–	–	–	–	–	–	–	–
	**PDC-373**	8	LCR-1 (*n* = 2)	–		–	–	–	–	–	–	–	–	–	–
ST395 (*n* = 1)	PDC-8	>128	OXA-1049	–	*∆*	*∆*	–	–	–	–	–	–	–	–	–
ST412 (*n* = 1)	PDC-1	4	–	–		–	L212P	E648K	–	–	–	–	–	S572N	–
ST500 (*n* = 1)	PDC-1	16	–	–	*∆*	*∆*	*∆*	–	–	–	–	–	–	–	–
ST534 (*n* = 3)	PDC-35	>128	PER-1	–		–	–	–	–	R132*fs*	–	R76L	–	–	–
		4	PER-1	–		–	–	–	–	–	–	R76L	–	–	–
		4	PER-1	–		–	–	–	–	*–*	–	R76L	–	–	R4RALP
ST560 (*n* = 1)	**PDC-145**	4	–	–		–	–	*∆*_1-150_	*∆*	*∆*	–	–	–	–	–
ST654 (*n* = 2)	PDC-3	8	NDM-1	–		–	–	–	–	–	–	–	–	A27T	–
		8	NDM-1, GES-9	–		S528A	–	–	–	–	–	–	–	A27T	–
ST664 (*n* = 2)	PDC-98	>128	PAC-1, OXA-10	L346MF533L	N688*fs*		–	–	–	–	–	–	–	–	–
ST675 (*n* = 1)	**PDC-346**	32	–	G63S R504C	–		K41*fs*	–	–	L45V R132*fs*	–	N787I	–	–	–
ST773 (*n* = 19)	PDC-16	16	NDM-1 (*n* = 8)	–	–		–	–	–	–	–	–	–	–	–
		16	NDM-1, CARB-2, OXA-10	–	–		–	–	–	–	–	–	–	–	–
		8	NDM-1	R504C	–		–	–	–	–	–	–	–	–	–
		8	NDM-1 (*n* = 5)	–	–		–	–	–	–	–	–	–	–	–
		8	NDM-1, VIM-4	–	–		–	–	–	–	–	–	–	–	–
		4	NDM-1 (*n* = 3)	–	–		–	–	–	–	–	–	–	–	–
		8	–	–	–		Y153S P154R S157 *fs*	–	–	–	–	–	–	–	–
ST981 (*n* = 1)	PDC-1	16	NDM-1	–	–		–	–	–	–	–	–	–	–	–
ST1047 (*n* = 3)	PDC-12	8	DIM-1, VEB-9	–	–		–	–	–	–	–	–	–	–	–
		8	IMP-1, OXA-10	–	–		–	–	–	–	–	–	–	–	–
		8	–	–	–		–	–	–	R132*fs*	–	–	–	–	–
ST1689 (*n* = 1)	PDC-1	64	–	–		*–*	G90*fs*	G685D	–	R132*fs*	–	–	–	–	–
ST1632 (*n* = 1)	**PDC-500**	16	–	–	*∆*	*∆*	*–*	274*Ins*_110 bp_	–	–	–	–	–	–	–
ST1716 (*n* = 1)	PDC-167	8	–	–	*∆*	*∆*	*∆*	L481F	–	–	–	–	–	T582S	–
ST2877 (*n = 1*)	PDC-8	16	–	–	*∆*	*∆*	*∆*	–	–	–	–	D121H	–	–	–
ST3302 (*n = 1*)	PDC-3	>128	–	–	–		–	–	–	–	R412L	–	–	–	–
ST3486 (*n = 1*)	PDC-3	4	–	–		–	–	–	–	–	–	–	–	A113T	–
ST3559 (*n = 1*)	**PDC-573**	64	–	T77A N427S R504C		–	–	S357F N375S T424*fs*	T114S	R132*fs*	–	–	–	–	–
ST4013 (*n = 1*)	**PDC-229**	16	–	G63S T267A R504C	K42*fs*		–	–	–	R132*fs*	–	*∆*	–	–	–
ST4049 (*n = 1*)	PDC-20	8	–	–	–		–	–	–	–	–	–	–	–	–
ST4156 (*n* = 1)	**PDC-293**	8	–	A244T	–		L209P	–	–	R132*fs*	A441V	F623L	*–*	*–*	*–*
ST4392 (*n = 1*)	PDC-19	16	–	–	–		–	–	–	–	–	–	–	–	–
ST4409 (*n = 1*)	**PDC-264**	4	–	F533L	–		–	–	–	R132*fs*	–	A653D	–	A27T G485S	–
ST4909 (*n = 1*)	**PDC-51**	128	–	G63D		–	–	T424*fs*	–	R132*fs*	–	D384N	V265E	–	G262E
ST4414 (*n = 1*)	PDC-3	8	VIM-1, OXA-2	–		–	–	–	–	R132*fs*	–	–	–	–	–
ST4516 (*n = 1*)	PDC-35	4	–	–	–		–	–	–	R132*fs*	–	–	–	–	–
ST4934 (*n = 1*)	**PDC-599**	>128	IMP-1, PAC-1, OXA-10	–		–	–	–	–	*–*	–	–	–	–	–
ST4936 (*n = 1*)	PDC-35														

^*a*^ A dash (-) indicates a wild-type protein. The absence of dash indicates that the protein PiuA or PiuD is absent; *fs* and *ins* indicate a frameshift and an insertion, respectively, and *∆* indicates the full deletion of the protein.

^*b*^ According to PubMed MLST scheme.

^*c*^ *Pseudomonas*-derived AmpC; values in bold indicate an extended-spectrum AmpC variant.

^*d*^ Orthologous proteins.

While nearly one-third (29.1%, *n* = 30) of cefiderocol-resistant strains produced NDM-1, their cefiderocol susceptibility ranged from an MIC of 4 to >128 mg/L, indicating that other factors may be influencing susceptibility. These factors could include the expression of additional ß-lactamases, with seven NDM-1 producer strains also found to carry either one (*n* = 4) or two additional ß-lactamases (*n* = 3), including PAC-1, OXA-10, LCR-1, CARB-2, PME-1, VEB-9, or GES-9 (Table 2; Table S2). Of the many MBL identified in the cefiderocol-resistant strains, the majority have previously been validated to cause cefiderocol resistance in engineered laboratory strains *in vitro* (22–24). An exception to this is four MBL enzymes (IMP-1, IMP-26, VIM-4, and AFM-5) whose role in cefiderocol resistance has not been previously confirmed (22–24). To evaluate if these MBLs are involved in conferring cefiderocol resistance, a broad-spectrum, zinc-chelating MBL inhibitor, dipicolinic acid, was used to inactivate these MBLs in the cefiderocol-resistant strains and evaluate cefiderocol susceptibility (Table 3).

**TABLE 3:** Impact of dipicolinic acid on cefiderocol susceptibility in MBL-producing *P. aeruginosa* clinical strains^*a*^

Clinical strain	Acquired β-lactamase	MIC of cefiderocol (mg/L)	MIC of cefiderocol (mg/L) / dipicolinic acid (100 mg/L)
CIP76110	Reference strain	0.125	0.125
PSA-19	**NDM-1**	32	0.25
9159	**NDM-1**/**VIM-4**	8	0.06
9611	**VIM-4**/OXA-641/CARB-2	8	1
11334	**IMP-1**/OXA-10	8	2
11558	**IMP-1**/PAC-1/OXA-10	>128	8
8610	**IMP-13**/CARB-2/OXA-4	4	1
9321	**IMP-13**/CARB-2/OXA-4	4	1
11485	**IMP-26/AFM-5**/ACA-7	16	0.5
11016	**VIM-1**/OXA-2	8	2
9350	**VIM-2**/GES-1/OXA-10	4	0.125
11165	**VIM-2**/VEB-9/OXA-520	4	1
11595	**VIM-2**/PER-1/OXA-4	4	0.25
10311	**DIM-1**/VEB-9	8	8

^*a*^ The metallo-β-lactamases are indicated in bold.

With the exception of the clinical strain producing DIM-1, for which no effect was expected based on previous *in vitro* observations, the addition of dipicolinic acid fully or partially restored cefiderocol susceptibility in these MBLs (NDM-1/VIM-4 and VIM-2/GES-1/OXA-10) producers (Table 3). These data support the role of VIM-2/GES-1/OXA-10 in cefiderocol resistance in clinical strains of *P. aeruginosa* (Table 3).

Among the 103 cefiderocol-resistant strains, there was a high proportion of clinical strains carrying ESBL genes (38.8%, *n* = 40), which represents a higher-than-average prevalence in clinical strains in France (25). Among the ESBL, Ambler Class A ESBLs were the most frequent, including SHV-2a (*n* = 1), PER-1 (*n* = 10), PME-1 (*n* = 1), VEB-1 (*n* = 1), VEB-9 (*n* = 8), VEB-20 (*n* = 1), GES-1 (*n* = 3), GES-9 (*n* = 3), GES-27 (*n* = 1), and ACA-7 (*n* = 1). These were followed by Ambler Class D ES-OXA enzymes (*n* = 12), among which OXA-19 was the most common variant (*n* = 5) (Table 2; Table S2). While the role of some of the ESBLs has been confirmed in cefiderocol resistance, this is not the case for all. To confirm their contribution to cefiderocol resistance, VEB-1, VEB-9, ACA-7, OXA-142, OXA-1049, and OXA-1170 (and others) were cloned into a pUCP24 plasmid and expressed in PAO1, followed by antibiotic susceptibility testing (Table 4). Data confirmed all these ESBL enzymes to confer 4-fold to 128-fold cefiderocol resistance, with particularly high resistance mediated by VEB-9 and VEB-1 (Table 4).

**TABLE 4:** Antibiotic susceptibility of PAO1 carrying recombinant ß-lactamase plasmids^*a*^

Strain	Phenotype	MIC (mg/L)									
		FDC	CAZ	CZA	CT	FEP	CFZ	AZM	IMP	IMR	MEM
PAO1(pUCP24)	Wild-type	0.125	2	2/4	≤0.5/4	1	≤0.5/0.5	4	1	≤0.5/4	≤0.5
PAO1 + VEB-1	ESBL	**8**	>64	8/4	>32/4	>32	2/2	>64	1	≤0.5/4	1
PAO1 + VEB-9^*b*^	ESBL	**16**	>64	8/4	>32/4	>32	2/2	>64	1	≤0.5/4	≤0.5
PAO1 + ACA-7^*c*^	ESBL	1	16	1/4	4/4	64	1/1	32	1	≤0.5/4	1
PAO1 + OXA-2	Narrow spectrum Oxacillinase	2	16	2/4	4/4	1	1/1	≤ 2	2	≤ 0.5/4	2
PAO1 + OXA-1049^*d*^	ESBL	**4**	>64	8/4	4/4	8	2/2	4	1	≤0.5/4	1
PAO1 + OXA-10	Narrow spectrum Oxacillinase	0.5	1	1/4	1/4	8	1/1	4	2	≤ 0.5/4	≤0.5
PAO1 + OXA-142^*e*^	ESBL	**4**	32	8/4	4/4	8	2/2	4	1	≤0.5/4	1
PAO1 + OXA-1170^*f*^	ESBL	**4**	16	8	2/4	4	1/1	8	1	≤0.5/4	1
PAO1 + OXA-641	Carbapenemase	1	16	4/4	16	8	2/2	16	2	0.5/4	1
PAO1 + OXA-677	Carbapenemase	0.25	2	1	0.5	1	≤0.5/0.5	<2	4	1	8
PAO1 + PAC-1	Cephalosporinase	**16**	>64	>32/4	>32/4	>32	2/2	4	1	≤0.5/4	1

^*a*^ FDC, cefiderocol; CAZ, ceftazidime; CZA, ceftazidime/avibactam; CT, ceftolozane/tazobactam; FEP, cefepime; CFZ, cefepime/zidebactam; AZM, aztreonam; IMP, imipenem; IMR, imipenem/relebactam; MEM, meropenem; ESBL, extended-spectrum ß-lactamase. The cefiderocol MIC values higher than the EUCAST clinical breakpoint (>2 mg/L) are indicated in bold.

^*b*^ VEB-9 is an I19V variant of VEB-1.

^*c*^ ACA-7 is a new variant of ACA-1 (89.0% identity of sequence).

^*d*^ OXA-1049 is a D150Y variant of OXA-2.

^*e*^ OXA-142 is an N74S, G171D variant of OXA-10.

^*f*^ OXA-1170 is a T117S, Y188F, E245G, S261N, and E275A variant of OXA-10.

In addition to ESBL enzymes, four highly cefiderocol-resistant clinical strains (CMI >128 mg/L) were found to carry a PAC-1 cephalosporinase in association with an OXA-10 oxacillinase or the MBL, IMP-1, or NDM-1 carbapenemases (26). As above, to explore the specific role of PAC-1 cephalosporinase in cefiderocol resistance, *bla*_PAC-1_ was cloned into PAO1 and was found to increase in cefiderocol MIC from 0.125 to 16 mg/L (Table 4). These data reveal that carbapenemases (particularly NDM-1), as well as class A and D ESBLs, significantly contribute as determinants of cefiderocol susceptibility.

### Intrinsic enzymatic resistance mechanisms in cefiderocol-resistant clinical strains

Among the 103 resistant strains, 30 different PDC sequences were identified. The most frequent variants were PDC-16 (19.4%, 20/103), PDC-35 (13.6%, 14/103), PDC-3 (12.6%, 13/103), PDC-11 (10.7%, 11/103), and PDC-8 (6.8%, 7/103). Genomic analysis of the PDCs and exclusion of natural polymorphisms revealed that 14 strains exhibited ESAC variants (13.6%), including mutations V211A (*n* = 5), E219K (*n* = 3), T70I (*n* = 2), G156D (*n* = 2), F121L (*n* = 1), and Δ291-293 (Table 5). To evaluate the contribution of these mutations, the different *bla*_PDC-1_ mutants were cloned into PAO1Δ*bla*_PDC-1_, and their impact on cefiderocol susceptibility was determined. The substitution E219K and the deletion Δ292-294, located in the Ω loop and the R2 domains of the proteins, respectively, led to the largest increase in cefiderocol MIC (from 0.5 to 4 mg/L) in comparison to the other substitutions, which resulted in MICs of 1 and 2 mg/L. In addition to cefiderocol, most of these mutations significantly affected susceptibility to ceftolozane/tazobactam, particularly E219K and T70I (Table 5), indicating a cross-resistance between ceftolozane/tazobactam and cefiderocol.

**TABLE 5:** Effects of PDC variants on cefiderocol susceptibility^*a*^^,^^*c*^

PDC variant	Mutation according to amino acid sequence of PDC-1 enzyme^*b*^	Mutation according to SANC position^c^	MIC (mg/L)						
			FDC	TZP	CAZ	CZA	CT	FEP	AZM
PDC variants identified in clinical strains with an ESAC activity									
PDC-51	R53Q, T79A, **V213A**	V211A	**4**	8/4	16	4/4	8/4	32	8
PDC-145	T79A, V179L, **V213A**, G365P	V211A	**4**	>256/4	>64	32/4	64/4	>32	>128
PDC-229	V19A, T79A, **V213A**, V330I, G365A	V211A	**64**	>256/4	>64	>64/4	>64/4	>32	128
PDC-230	V19A, T79A, **V213A**, G365A	V211A	**32**	>256/4	>64	64/4	>64/4	>32	>128
PDC-264	R53Q, T79A, **F121L**	F121L	**16**	256/4	>64	16/4	>64/4	32	128
PDC-293	T79A, G365, **Δ292-294**	Δ291-293	**8**	32/4	64	4/4	4/4	>32	8
PDC-334	**T70I**, T79A	T70I	**4**	64/4	>64	64/4	>64/4	>32	128
PDC-346	R53Q, T79A, **G157D**,	G156D	**32**	128/4	>64	>64/4	>64/4	>32	>128
PDC-373	**T70I**, T79A, V179L, **E221K**, G365A	T70I + E219K	**4**	8/4	>64	>64/4	>64/4	32	64
PDC-500	G1D, T79A, **G157D**, V179L, G365P	G156D	**64**	32/4	>64	64/4	64/4	16	32
PDC-515	R53Q, T79A, **E221K**, P248L	E219K	**4**	32/4	>64	>64/4	>64/4	32	64
PDC-573	G1D, T79A, A130T, V179L, **E221K**, G365A	E219K	**4**	32/4	>64	64/4	>64/4	32	64
PDC-599	**V213A**	V211A	**4**	>256/4	>64	>64/4	64/4	>32	>128
Cloning of variants in PAO1Δ*bla*_PDC-1_^*d*^									
PDC-1	–*^e^*	–	0.5	≤4/4	2	2/4	≤0.5/4	2	4
–	V213A	V211A	1	64/4	64	4/4	4/4	8	32
–	F121L	F121L	1	32/4	32	2/4	8/4	8	16
–	G157D	G156D	2	8/4	32	8/4	32/4	4	8
–	T70I	T70I	2	8/4	64	8/4	64/4	16	16
–	E221K	E219K	**4**	16/4	>64	8/4	>64/4	8	32
–	Δ292-294	Δ291-293	**4**	16/4	32	4/4	4/4	>32	16

^*a*^ Amino acids indicated in bold correspond to ESAC variants. The cefiderocol MIC values higher than the EUCAST clinical breakpoint (>2 mg/L) were indicated in bold.

^*b*^ Positions of mutated amino acid residues refer to the mature PDC-1 sequence (accession number WP_003101289.1) (lacking the 26 amino acid long signal peptide required for export to the periplasm).

^*c*^ Positions of mutated amino acid residues refer to the SANC system based on AmpC enzyme from *Enterobacter cloacae *strain P99.

^*d*^ Amino acid substitutions or deletion D292-294 were generated from *bla*_PDC-1 _gene by mutagenesis, cloned into pUCP-24, and introduced into the mutant PAO1Δ*bla*_PDC-1._

^*e*^ –, wild-type sequence of PDC.

### Outer membrane *tonB*-dependent siderophore transporters and their regulators

In addition to cefiderocol resistance by β-lactam-mediated hydrolysis, various mutations that impact active tonB-dependent receptors and periplasmic uptake of cefiderocol cause resistance by limiting uptake. Such mutations have been reported both in the transporters themselves (particularly PiuA [or ortholog PiuD] and PirA) and in the regulators of these transporters (8, 10). Genome analysis of the 103 cefiderocol resistance strains found 40 strains (38.8%) to have a non-synonymous nucleotide polymorphism (SNPs) in either the TonB-dependent transporter or their known regulators, and these mutations were particularly frequent and often multiple in strains with high cefiderocol MIC levels (>16 mg/L) (Table 2; Table S2). Regarding TonB-dependent transporters, SNPs were identified in *piuA/piuD* (*n* = 11, 10.7%), *pirA* (*n* = 9, 8.7%), *fpvB* (*n* = 9, 8.7%), *piuC* (*n* = 8, 7.8%), *fecA* (*n* = 8, 7.8%), *fptA* (*n* = 5, 4.9%), *pfeA* (*n* = 5, 4.9%), and *fiuA* (*n* = 3, 2.9%), although association with cefiderocol resistance is not known in all cases. Most of these identified mutations resulted in protein truncation, aberrant sequences, or complete gene deletion and thus predicated the loss of transporter function. While deletions of the PiuA hydroxamate-type ferrisiderophore receptor and the PirA ferric enterobactin receptor have previously been shown to impact cefiderocol susceptibility, we further evaluated the impact of deleting *piuC*, *fptA*, *pfeA*, and *fpvA* in the PAO1 strain on cefiderocol activity (Table S3). This evaluation found that neither *fptA* nor *pfeA* played a major role in cefiderocol transport and that loss of *fpvA* led to sensitization to cefiderocol (likely due to increased iron stress by inability to take up pyoverdin). Interestingly, deletion of *piuC*, which encodes an iron-dependent oxygenase TonB-dependent receptor, did result in a significant increase in the cefiderocol MIC (from 0.125 to 2 mg/L) and may thus be a genetic factor regulating cefiderocol resistance in clinical strains. Regarding the transcriptional regulators of the TonB-dependent transporter, SNPs were identified in *pirR* (*n* = 16, 15.5%) and *pirS* (*n* = 3, 2.9%). PirR is the response regulator belonging to the two-component system PirRS that modulates the expression of both the PirA and PiuA (PiuD) transporter and was the most frequently mutated transport-related locus in the cefiderocol-resistant clinical strains. Interestingly, except for one strain that has a deletion of one nucleotide in *pirR* gene, all the strains have a frameshift mutation (R132*fs*) occurring in a region with a succession of seven guanine residues from nucleotides 389 to 395 (amino acid positions 132 or 133). The cefiderocol MICs of strains harboring the *pirR* frameshift ranged from 4 to 128 mg/L (Table 2). Of these *pirR* frameshift mutants, only one strain that exhibited a cefiderocol MIC of 8 mg/L lacked any acquired β-lactamase or ESAC variant (ST1689, PDC-1), suggesting an important role of PirR function in clinical susceptibility to cefiderocol. Deletion of the *pirR* gene in the strain PAO1 confirmed that loss of function of PirR leads to a significant reduction in cefiderocol activity (4-fold increase in MIC) (Table S3).

## DISCUSSION

In this nationwide study, we analyzed 103 cefiderocol-resistant *P. aeruginosa* clinical isolates collected over a 4-year period and provided a comprehensive overview of the epidemiology and mechanisms underlying resistance to this last-resort siderophore cephalosporin. Cefiderocol-resistant isolates exhibited extensive multidrug resistance, with high rates of resistance to β-lactams, carbapenems, and β-lactam/β-lactamase inhibitor combinations (ceftolozane/tazobactam, ceftazidime/avibactam, and imipenem/relebactam). This was consistent with the high proportion of strains producing carbapenemases and/or an ESBL (72.8%, *n* = 75) in our collection. These findings align with recent epidemiological data showing that cefiderocol non-susceptibility is enriched among ceftolozane/tazobactam-resistant (8.3%, 95% CI 4.1%–16.0%) and NDM-producing isolates (22.9%, 95% CI 8.1%–49.7%) (27). In this context, colistin remained the most consistently active antibiotic, highlighting its role as a last-line therapeutic option.

Cefiderocol resistance has been reported across multiple STs, including high-risk clones such as ST111, ST175, ST235, and ST308, without any clear lineage-specific association (4, 18, 28–30). Together with our data, this supports a polyclonal emergence of resistance driven by diverse chromosomal and acquired mechanisms rather than clonal dissemination.

Although cefiderocol was initially considered stable against metallo-β-lactamases, a recent molecular study has demonstrated that NDM-1 enzyme efficiently hydrolyzes cefiderocol, whereas VIM-2 and IMP-1 exhibited limited activity (31). Several studies have reported reduced susceptibility or resistance in MBL-producing *P. aeruginosa*, particularly when additional resistance mechanisms are present (23, 24, 28, 32). Our data confirm that transferable β-lactamases play a major role in reducing cefiderocol susceptibility (Tables 2 and 3; Table S1). MBLs, especially NDM-1 (29.1%, *n* = 30), were highly prevalent and strongly associated with elevated MICs (Table S2). Among strains with MIC values higher than 8 mg/L (*n* = 41), 39.5% (*n* = 17) produced NDM-1. These findings highlight the clinical importance of NDM producer strains detection, as cefiderocol use should be considered with caution in this context. Beyond carbapenemases, a diversity of class A ESBLs, particularly VEB-9 and PER-1, as well as class D ESBLs such as OXA-19, were frequently identified and contributed to reduced susceptibility to cefiderocol, as confirmed by cloning experiments. Notably, the acquired cephalosporinase PAC-1 emerged as a potent determinant, conferring high-level resistance to cefiderocol, as well as ceftolozane/tazobactam and ceftazidime/avibactam. These results suggest that PAC-1 may represent an underappreciated contributor to resistance against siderophore cephalosporins (26).

In addition to acquired enzymes, mutations affecting the Ω-loop region of the chromosomal β-lactamase PDC, particularly E219K, have been associated with reduced susceptibility to several β-lactams including ceftolozane/tazobactam, ceftazidime/avibactam, and cefiderocol (23, 33). We confirm the major impact of this substitution and identify additional mutations, including V211A and a Δ291–293 deletion, further expanding the spectrum of PDC mutations associated with decreased cefiderocol susceptibility. The Δ291–293 deletion is located within the R2 binding region, which is critical for the recognition of bulky cephalosporins, and likely enhances hydrolytic activity. Recently, it was shown that the mutation E219K affecting the Ω-loop region is associated with increased catalytic turnover, whereas substitution L293P in R2-loop (Helix H10) enhanced substrate binding affinity (34). Although the substitution E219K remains rare among clinical strains (35 PDC sequences of the 686 variants), its strong functional impact supports the need for molecular surveillance of PDC variants associated with cefiderocol resistance (35, 36).

Alterations in siderophore-mediated uptake pathways also emerged as a key mechanism. Loss-of-function mutations affecting TonB-dependent receptors such as PiuA/PiuD, PirA, and associated proteins have been reported in both clinical isolates and experimental models (8, 10, 30, 37–39). In our study, mutations were most frequently identified in the response regulator PirR, a finding that has been only rarely described (30, 39). A recurrent frameshift mutation, R132fs in the *pirR* gene, was observed across multiple genetic backgrounds, suggesting convergent evolution. This particular frameshift in the *pirR* gene has also been reported in strains from a general hospital in Houston, Texas, not previously exposed to cefiderocol (30). Functional analysis confirmed that loss of PirR significantly reduces cefiderocol susceptibility, reinforcing the central role of impaired iron uptake.

High levels of resistance to cefiderocol (MIC >16 mg/L) were consistently associated with the combination of at least two mechanisms involving β-lactamases together with alterations in siderophore transport systems. This highlights the synergistic interaction between enzymatic degradation and reduced antibiotic uptake.

Mutations in *ftsI*, encoding PBP-3, the primary target of cefiderocol, were more frequently observed among isolates with MIC values higher than 16 mg/L (Table 2; Table S2). However, the functional impact of the nine different mutations identified in 28 strains (27.1%) was not explored in this study. While some PBP-3 substitutions, such as R504C and F533L, identified in 10.7% (*n* = 11) and 20.9% (*n* = 3) of our collection, respectively, have been reported to increase susceptibility to cefiderocol, suggesting that target modifications may enhance antibiotic binding rather than confer resistance (40). An evolutionary study involving three clinical strains with distinct genetic backgrounds (ST111, ST175, and ST235) revealed the frequent emergence of PBP-3 mutations (A421V, L506P, and R551C) following cefiderocol exposure. These results suggest that PBP-3 mutations alone are not sufficient to confer cefiderocol resistance but may require additional resistance determinants (production of β-lactamases, PDC variants, and/or alteration of siderophore transporters) and act synergistically with other resistance mechanisms to contribute to cefiderocol resistance (12).

Finally, although cefiderocol is relatively stable to efflux, overexpression of MexAB-OprM has been associated with modest 2-fold increases in MIC (7, 22). In our collection, 48.5% (*n* = 50) of strains carried inactivating mutations in at least one of the three major regulators of the efflux pump MexAB-OprM (*mexR*, 13.7%, *n* = 15; *nalC, 5.8%, n* = 6; *nalD*, 33.0%, *n* = 34). As *nalD* mutations are generally less frequent than alterations in *mexR* or *nalC*, their contribution is likely secondary (41). However, indirect effects on cefiderocol susceptibility cannot be excluded, given the complex regulatory networks of *P. aeruginosa* (42).

Overall, our study demonstrates that cefiderocol resistance in *P. aeruginosa* is multifactorial and results from a complex interplay between acquired β-lactamases, chromosomal mutations, and alterations in iron uptake pathways. The absence of clonal clustering and the diversity of resistance mechanisms highlight the difficulty of predicting cefiderocol susceptibility in clinical practice. These findings underscore the need for continued surveillance and systematic susceptibility testing, particularly in strains producing ESBL and carbapenemases.

## MATERIALS AND METHODS

### Strain collection and antibiotic susceptibility testing

From the FNRC for Antibiotic Resistance (RA) collection, we selected all *P. aeruginosa* clinical strains resistant to cefiderocol based on the current EUCAST breakpoints (MIC >2 mg/L) (43). A total of 103 strains, isolated from 61 French hospitals between 2021 and 2024, were selected for the study. Cefiderocol MIC values were determined using broth microdilution in iron-depleted cation-adjusted Mueller-Hinton Broth (ID-CAMHB), following EUCAST recommendations, with the exception of the chelation contact time. Briefly, chelation of the cation MHB (Becton Dickinson) was conducted for 12 h using CHELEX resin (Bio-Rad, 10 g/100 mL). The resulting mixture underwent filtration and supplementation with Ca^2+^ (22.5 μg/mL), Mg^2+^ (11.3 μg/mL), and Zn^2+^ (0.7 μg/mL). Titrated cefiderocol powder (CliniSciences, Nanterre, France) was used for testing. Susceptibility testing for other antibiotics, including piperacillin/tazobactam, ceftazidime, ceftazidime/avibactam, ceftolozane/tazobactam, cefepime, aztreonam, imipenem, imipenem/relebactam, meropenem, and colistin, was performed using broth microdilution in CA-MHB (Thermo Fisher Scientific) with customized Sensititre microplates (Thermo Fisher Scientific). MIC values were interpreted according to EUCAST 2025 breakpoints (43). To evaluate the impact of selected metallo-β-lactamases on cefiderocol susceptibility, the MIC of cefiderocol was determined in the presence of 100 mg/L of dipicolinic acid.

### Whole genome sequencing and bioinformatic analysis

Total DNA from cefiderocol-resistant strains was extracted using the PureLink Genomic DNA Kit (Thermo Fisher Scientific). DNA libraries were constructed using the Nextera XT DNA (Illumina), and sequencing of 150 bp paired-ends was performed on the Illumina NextSeq 500 sequencer (PibNet platform, Institut Pasteur, Paris). Data were processed using bcl2fastQ Conversion Software (v2.20, Illumina) to achieve an average depth of at least 80 for each nucleotide. DNA sequences (from 4,419,590 to 6,752,280 reads) were trimmed using FastP software (v0.23.2), and contig assembly and annotation were conducted using shovill-SPAdes (v3.15.4) and Prokka (v1.14.6) software, respectively. ST was determined using Pasteur’s MLST schema. Resistance genes were identified using the FNRC-RA pipeline, which is based on the NDARO and CARD databases. Mutations in genes previously associated with alteration of cefiderocol activity or involved in iron transport or regulation (*bla*_PDC_, *piuA, piuC, piuD, pirA, pirR, pirS, fptA, fpvB, pfeA, fiuA, fecA, ftsI, mexR, nalB,* and *nalC*) were identified by comparing the sequences of cefiderocol-resistant strains to those of reference strains PAO1, PA14, CF39S, and a large collection of clinical strains susceptible to cefiderocol (MIC ≤0.5 mg/L) (*n* = 485 strains) to suppress natural polymorphisms using CLC Genomic Workbench (v10.1.1) (44). Mutations found to be identical in more than two strains belonging to different STs were considered polymorphisms and were filtered out. The CF39S strain was chosen as the reference strain because the *piuD* gene (orthologous to *piuA*) is absent from the genomes of PAO1 and PA14. All genome sequences are available at accession number PRJNA1190866 (BioProject - NCBI).

### Cloning of interest genes into the pUCP24 plasmid

Total DNA from clinical strains was extracted from a bacterial colony using the PureLink Genomic DNA Kit (Thermo Fisher Scientific). Selected genes were amplified with specific primers and cloned into a linearized vector using the NEBuilder HiFi DNA Assembly Kit (New England Biolabs), following the manufacturer’s instructions. These were then cloned into the shuttle vector pUCP24 via restriction enzymes XbaI NEB (for genes *bla*_OXA_, *bla*_PAC-1_, and *bla*_VEB_), or HindIII and EcoRI (for the *bla*_PDC_- genes). Mutagenesis of gene *bla*_PDC-1_ was performed using the Q5 site-directed mutagenesis kit (New England Biolabs). Recombinant vectors were transferred into the PAO1 strain or its derived mutant PAO1Δ*bla*_PDC-1_ by electroporation (MicroPulser Electroporator, Bio-Rad). Transformants were selected on Mueller-Hinton agar medium supplemented with gentamicin (50 µg/mL).

### Deletion of genes in strain PAO1

Single knockout mutants in the *piuC*, *pirR*, *fptA*, *fpvA*, and *pfeA* genes were constructed in the reference strain PAO1 using overlapping PCR and recombination events, as previously described (45). Briefly, fragments of −500 bp flanking the target genes were amplified with specific primers. These amplicons were then used as templates for overlapping PCR with external primers to amplify the recombinant fragments. The resulting amplicons were cloned into a linearized vector using the NEBuilder HiFi DNA Assembly Kit (NEB) and subsequently subcloned into the suicide vectors pKNG101 or pFOG and *E. coli* CC118λ*pir* as BamHI-HF or BamHI-EcoRI fragments. The recombinant vectors were then transferred into the PAO1 strain by conjugation with the pRK2013 vector, and deletion mutants were selected on *Pseudomonas* isolation agar supplemented with 2,000 µg/mL streptomycin. Deletion mutants were then isolated on minimum M9 supplemented with 5% sucrose. Both strands of the DNA were sequenced to confirm the deletion.

## SUPPLEMENTAL MATERIAL

Supplemental material aac.00520-26-s0001.pdf Tables S1 to S3.

ASM does not own the copyrights to Supplemental Material that may be linked to, or accessed through, an article. The authors have granted ASM a non-exclusive, world-wide license to publish the Supplemental Material files. Please contact the corresponding author directly for reuse.
