# Supplementary material for: Mechanisms involved in cefiderocol resistance in French Pseudomonas aeruginosa clinical strains
Source: Antimicrob Agents Chemother. 2026 Jul 10;70(8):e00520-26. doi: 10.1128/aac.00520-26 (PMC13435742; doi:10.1128/aac.00520-26)
Supplement: Supplementary file 1 [file aac.00520-26-s0001.pdf]

TABLE S1 Antibiotic susceptibility of 103 clinical strains of *Pseudomonas aeruginosa* included in the study.

Strains	MIC (mg/L)												
	TZP	ATM	CAZ	FEP	CZA	C/T	FDC	IMP	IPR	MEM	AKN	CIP	CST
9124	16/4	8	>64	32	>64/4	64/4	>128	4	2/4	8	>64	≤0.25	1
9556	>64/4	>64	>64	>64	>64/4	>64/4	>128	16	8/4	>64	>64	>4	1
9898	>64/4	64	>64	>64	>64/4	>64/4	>128	16	4/4	16	>64	≤0.25	1
10893	>64/4	32	>64	>64	>64/4	>64/4	>128	>64	>64/4	>64	>64	>4	2
11558	>64/4	64	>64	>64	>64/4	>64/4	>128	>64	>64/4	>64	>64	>4	2
11596	>64/4	>64	>64	>64	>64/4	>64/4	>128	16	8/4	32	32	>4	1
SD974-21	>64/4	64	>64	>64	>64/4	>64/4	>128	8	4/4	64	>64	>4	1
NIC-15	≤4/4	8	4	2	4/4	1/4	>128	2	≤0.5/4	≤0.5	2	≤0.25	2
11504	>64/4	>64	>64	>64	>64/4	>64/4	128	32	8/4	32	>64	>4	2
CHA-08	32/4	4	16	32	2/4	2/4	128	8	2/4	8	8	>4	≤0.5
9104	>64/4	>64	>64	>64	>64/4	>64/4	64	16	4/4	8	32	>4	1
9246	32/4	32	>64	16	64/4	64/4	64	1	2/4	16	8	>4	1
10088	16/4	8	>64	8	16/4	8/4	64	1	≤0.5/4	2	2	>4	1
10284	>64/4	>64	>64	>64	64/4	>64/4	32	64	8/4	32	>64	>4	1
10326	>64/4	>64	>64	>64	>64/4	>64/4	32	8	8/4	32	>64	1	1
PSA-19	>64/4	64	>64	> 64	> 64/4	>64/4	32	> 64	>64/4	>64	>64	0.5	1
10002	>64/4	16	>64	>64	>64/4	>64/4	16	>64	>64/4	>64	>64	>4	2
10340	>64/4	>64	>64	>64	16/4	32/4	16	32	8/4	32	>64	4	1
10391	>64/4	64	>64	32	16/4	8/4	16	32	4/4	16	8	≤0.25	2
10615	>64/4	2	>64	>64	>64/4	>64/4	16	>64	>64/4	>64	>64	>4	2
10840	>64/4	8	>64	>64	>64/4	>64/4	16	>64	>64/4	>64	>64	>4	1
10992	32/4	>64	>64	>64	64/4	>64/4	16	8	1/4	8	>64	>4	8
11095	>64/4	≤2	>64	>64	>64/4	>64/4	16	>64	>64/4	>64	>64	>4	1

11191	>64/4	>64	>64	>64	>64/4	>64/4	16	>64	>64/4	>64	>64	>4	2
11200	>64/4	32	2	64	1/4	8/4	16	8	4/4	32	4	≤0.25	1
11232	>64/4	16	>64	>64	>64/4	>64/4	16	>64	>64/4	>64	>64	>4	1
11240	>64/4	4	>64	>64	>64/4	>64/4	16	>64	>64/4	>64	>64	>4	1
11260	>64/4	4	>64	>64	>64/4	>64/4	16	>64	>64/4	>64	>64	>4	1
11268	>64/4	8	>64	>64	>64/4	>64/4	16	>64	>64/4	>64	>64	>4	1
11273	>64/4	>64	>64	32	16/4	>64/4	16	8	1/4	4	4	≤0.25	1
11286	>64/4	32	>64	64	>64/4	>64/4	16	>64	>64/4	>64	>64	>4	1
11345	>64/4	16	>64	>64	>64/4	>64/4	16	>64	>64/4	>64	>64	>4	1
11359	>64/4	64	>64	>64	>64/4	>64/4	16	>64	>64/4	>64	>64	>4	1
11384	>64/4	≤2	>64	>64	>64/4	>64/4	16	>64	>64/4	>64	>64	>4	1
11402	>64/4	>64	>64	64	64/4	8/4	16	2	≤0.5/4	2	2	0.5	1
11462	>64/4	>64	>64	>64	>64/4	>64/4	16	32	8/4	32	32	>4	1
11485	>64/4	32	>64	>64	>64/4	>64/4	16	>64	>64/4	>64	32	>4	1
11486	>64/4	>64	>64	>64	>64/4	>64/4	16	>64	>64/4	>64	>64	>4	1
11531	64/4	>64	32	32	16/4	4/4	16	32	8/4	16	8	≤0.25	1
11533	>64/4	64	>64	>64	>64/4	32/4	16	8	2/4	8	>64	>4	1
11586	>64/4	≤2	>64	>64	>64/4	>64/4	16	>64	>64/4	>64	>64	>4	1
2963	>64/4	64	>64	>64	>64/4	64/4	8	32	16/4	32	32	>4	2
8502	>64/4	16	>64	>64	>64/4	>64/4	8	>64	>64/4	>64	>64	>4	2
9023	>64/4	>64	64	32	16/4	4/4	8	16	2/4	32	16	0.5	2
9148	>64/4	4	>64	>64	>64/4	>64/4	8	>64	>64/4	>64	>64	>4	2
9159	>64/4	8	>64	>64	>64/4	>64/4	8	>64	>64/4	>64	>64	>4	1
9460	>64/4	4	>64	>64	>64/4	>64/4	8	>64	>64/4	>64	>64	>4	≤0.5
9611	>64/4	>64	>64	>64	>64/4	>64/4	8	>64	>64/4	>64	4	>4	1
9731	>64/4	>64	>64	>64	64/4	>64/4	8	8	1/4	8	8	>4	1
9917	>64/4	16	>64	>64	>64/4	>64/4	8	>64	>64/4	>64	>64	>4	2
10070	>64/4	64	>64	64	>64/4	64/4	8	16	8/4	16	32	>4	1
10311	>64/4	>64	>64	>64	>64/4	>64/4	8	2	2/4	32	>64	>4	1

10650	64/4	64	16	16	16/4	2/4	8	32	8/4	32	4	0.5	2
10845	>64/4	>64	>64	>64	32/4	16/4	8	32	16/4	32	16	>4	1
10900	>64/4	4	>64	>64	>64/4	>64/4	8	>64	>64/4	>64	>64	>4	1
10984	>64/4	>64	>64	>64	>64/4	>64/4	8	>64	>64/4	>64	>64	>4	1
11016	>64/4	16	>64	>64	>64/4	>64/4	8	16	16/4	32	16	4	0.5
11052	>64/4	64	>64	>64	>64/4	>64/4	8	>64	>64/4	>64	>64	>4	2
11119	>64/4	64	>64	64	16/4	>64/4	8	32	4/4	8	16	0.5	1
11173	>64/4	16	>64	>64	>64/4	>64/4	8	>64	>64/4	>64	>64	>4	1
11282	32/4	32	>64	32	>64/4	>64/4	8	4	2/4	8	32	>4	1
11334	>64/4	32	>64	>64	>64/4	>64/4	8	>64	>64/4	>64	>64	>4	1
11393	>64/4	16	>64	>64	>64/4	>64/4	8	>64	>64/4	>64	>64	>4	2
11433	32/4	>64	>64	>64	64/4	>64/4	8	16	4/4	16	8	>4	1
COR-15	32/4	8	64	>64	4/4	4/4	8	≤ 0.5	≤0.5/4	≤0.5	2	≤0.25	2
LIE-07	>64/4	>64	>64	64	32/4	8/4	8	1	≤0.5/4	1	16	0.5	2
LYO-09	>64/4	>64	>64	>64	> 64/4	> 64/4	8	8	2/4	8	32	>4	1
SD93-04	16/4	32	>64	16	64/4	64/4	8	1	1/4	16	>64	>4	1
6598	>64/4	32	>64	64	>64/4	32/4	4	2	0.5/4	4	16	>4	2
8014	>64/4	>64	>64	>64	>64/4	>64/4	4	32	8/4	16	16	>4	0.5
8610	>64/4	16	>64	>64	>64/4	>64/4	4	>64	>64/4	>64	>64	>4	4
8643	8/4	>64	>64	>64	32/4	>64/4	4	2	0.5/4	0.25	8	≤0.25	1
8808	>64/4	>64	>64	>64	>64/4	>64/4	4	>64	4/4	16	32	>4	2
8928	>64/4	>64	64	16	16/4	2/4	4	>64	4/4	16	4	>4	2
9063	≤4/4	>64	>64	>64	8/4	8/4	4	16	4/4	4	>64	4	1
9321	>64/4	16	>64	>64	>64/4	>64/4	4	>64	>64/4	>64	>64	>4	2
9350	>64/4	16	>64	64	32/4	>64/4	4	64	64/4	64	>64	>4	1
9484	32/4	>64	>64	>64	>64/4	>64/4	4	16	4/4	16	64	>4	2
9579	8/4	64	>64	32	>64/4	>64/4	4	2	2/4	16	64	>4	2
9769	>64/4	>64	>64	>64	64/4	>64/4	4	16	2/4	16	8	>4	8
10061	64/4	>64	>64	64	>64/4	>64/4	4	1	≤0.5/4	2	4	0.5	1

10355	>64/4	≤4	>64	>64	>64/4	>64/4	4	>64	>64/4	>64	>64	>4	2
10385	64/4	64	>64	64	64/4	>64/4	4	32	16/4	32	>64	>4	2
10438	32/4	64	>64	32	64/4	>64/4	4	4	1/4	2	4	≤0.25	2
11512	>64/4	>64	>64	>64	64/4	64/4	4	8	2/4	8	32	0.5	>16
10539	64/4	>64	>64	>64	>64/4	>64/4	4	16	4/4	64	>64	>4	16
10562	>64/4	>64	>64	64	32/4	>64/4	4	16	2/4	16	32	>4	1
10630	>64/4	>64	>64	>64	32/4	64/4	4	32	8/4	16	32	4	1
10953	>64/4	>64	>64	64	32/4	>64/4	4	2	≤0.5/4	8	32	4	2
10976	>64/4	32	64	64	4/4	4/4	4	16	2/4	8	>64	>4	2
11060	>64/4	4	>64	>64	>64/4	>64/4	4	>64	>64/4	>64	>64	>4	1
11087	64/4	16	>64	64	16/4	>64/4	4	16	8/4	16	>64	>4	1
11164	>64/4	>64	8	>64	8/4	32/4	4	32	16/4	>64	32	4	1
11165	8/4	8	16	32	4/4	8/4	4	16	4/4	32	>64	2	≤0.5
11203	32/4	>64	>64	>64	64/4	>64/4	4	>64	>64/4	>64	8	>4	≤0.5
11218	>64/4	>64	>64	>64	>64/4	>64/4	4	16	4/4	8	32	≤0.25	1
11221	16/4	16	4	8	4/4	1/4	4	2	≤0.5/4	1	4	1	1
11315	>64/4	64	>64	>64	>64/4	>64/4	4	>64	>64/4	>64	>64	>4	2
11371	64/4	>64	>64	>64	32/4	>64/4	4	4	≤0.5/4	8	>64	>4	>16
11549	32/4	8	>64	32	4/4	>64/4	4	8	2/4	8	>64	>4	2
11595	>64/4	>64	>64	>64	>64/4	>64/4	4	16	16/4	8	>64	>4	1
AMI-01	32/4	64	>64	32	> 64/4	> 64/4	4	4	2/4	16	4	4	1
BET-15	8/4	16	4	2	4/4	2/4	4	8	≤ 0.5/4	8	2	≤0.25	1

TZP, piperacillin/tazobactam ; ATM, aztreonam ; CAZ, ceftazidime ; FEP, cefepime ; CZA, ceftazidime/avibactam ; C/T, ceftolozane/tazobactam ; FDC, cefiderocol ; IMP, imipenem ; IPR, imipenem/relebactam ; MEM, meropenem ; AKN, amikacin, CIP, ciprofloxacin ; CST, colistin.

TABLE S2 Resistome analysis of 103 cefiderocol-resistant clinical *Pseudomonas aeruginosa* strains

Strain	origin	Cefiderocol MIC (mg/L)	ExoS	ST type	Intrinsic cephalosporinase, PDC and its regulators						Efflux Pump MexAB-OprM					PBP-3								
					PDC ESAC variant	PBP-4	AmpD	AmpDh2	AmpDh3	AmpR	MexA	MexB	MexR	NalC	NalD	G63D/ G63S	T77A	A244T	T267A	L346M	N427S	R504C	G531D	F533L
9124	Digestive	>128	■	395																				
9556	Blood	>128	■	664							■				■					■				■
9898	Respiratory	>128	■	260																				
10893	Blood	>128		308											■									
11558	Urine	>128		4936											■									
11596	Blood	>128		534			■																	
SD974-21	Blood	>128	■	664							■				■					■				■
NIC-15	Respiratory	>128	■	3486											■									
11504	Bone	128		357					■						■									
CHA-08	Respiratory	128	■	4414							■	■				■								
9104	Respiratory	64	■	4013	■	■	■				■	■					■				■	■		
9246	Other	64		1632	■	■							■											
10088	Blood	64	■	244																				
10284	Respiratory	32	■	274	■	■	■					■					■				■	■		
10326	Respiratory	32	■	675	■	■				■	■					■						■		
PSA-19	Urine	32	■	274								■	■											
10002	Urine	16		773											■									
10340	Respiratory	16	■	4049								■				■			■			■		
10391	Blood	16	■	500			■																	
10615	Urine	16		773																				
10840	Urine	16		235			■																	
10992	Urine	16	■	179									■									■		
11095	Other	16		773																				
11191	Blood	16	■	233											■							■		
11200	Screening	16		253																				
11232	Urine	16		308											■									
11240	Skin	16		773																				
11260	Urine	16		1047																				
11268	Screening	16		773										■										
11273	Blood	16	■	4409	■					■														
11286	Blood	16		357					■						■									
11345	Screening	16		773										■										
11359	Screening	16		773									■									■		
11384	Screening	16		773																				
11402	Bone	16	■	3302			■						■											
11462	Respiratory	16		357					■						■									
11485	Blood	16		235											■									
11486	Respiratory	16		308											■									
11531	Respiratory	16	*	1716			■																	
11533	Blood	16		235			■																■	
11586	Urine	16		773																				
2963	Blood	8	■	233											■									
8502	Urine	8		773											■									
9023	Respiratory	8	■	2877			■								■									
9148	Respiratory	8		773																				
9159	Urine	8		773																				
9460	Bone	8		773																				
9611	Blood	8	■	175						■														
9731	Bone	8	■	179									■									■		
9917	Skin	8		773										■										
10070	Screening	8	■	111																				
10311	Bone	8		1047											■									
10650	Respiratory	8		313									■	■										
10845	Respiratory	8		4392														■						
10900	Screening	8		773																				
10984	Screening	8		357					■						■									
11016	Urine	8		4516									■											
11052	Urine	8	■	654											■									
11119	Skin	8	■	1689																				
11173	Urine	8	■	654											■									
11282	Respiratory	8		235									■											

Strain	PiuA/ piuD				PiuC				PirA								PirR			PirS		FptA					FpvB								PfeA					FiuA			FecA							
	PiuA abberant	PiuA T176A	PiuD S528A	PiuA and PiuD truncated	Abberant protein	Truncated protein	L209P	L212P	Abberant protein	Truncated protein	G363S	S357F	N375S	L481F	E648K	G685D	Abberant protein	Truncated protein	L45V	Truncated protein	P114S	Truncated protein	T68I	R412L	L439R	A441V	Abberant protein	Truncated protein	R76L	D121H	D384N	F623L	A653D	N787I	Truncated protein	Abberant protein	S2P	G262E	T654I	Abberant protein	V265E	M670I	Truncated protein	A27T	A113T	E710D	H373R	G485S	S572N	T582S
9124				■																																														
9556	■																																																	
9898																	■																																	
10893																																																		
11558																																																		
11596																	■												■																					
SD974-21	■																																																	
NIC-15																								■																										
11504																																																		
CHA-08									■								■														■							■			■									
9104									■			■	■				■				■																													
9246					■											■	■																													■	■			
10088	■																																									■								
10284									■								■				■															■														
10326					■												■		■															■																
PSA-19											■																																				■			
10002																																																		
10340	■																■											■																						
10391				■		■																																												
10615																																																		
10840																																																		
10992																							■																											
11095																																																		
11191																																																		
11200																	■																																	
11232																																																		
11240																																																		
11260																																																		
11268																																																		
11273																																																		
11286																																																		
11345																																																		
11359																																																		
11384																																																		
11402				■		■																								■																				
11462																																																		
11485																																																		
11486																																																		
11531				■						■																																								
11533																																																		
11586																																																		
2963																																																		
8502																																																		
9023				■		■								■																																				■
9148																																																		
9159																																																		
9460																																																		
9611																																																		
9731																																																		
9917																																																		
10070																									■																									
10311																																																		
10650		■															■																																	
10845							■										■									■						■																		
10900																																																		
10984																																																		
11016																	■																																	
11052			■																																									■						
11119																	■																																	
11173																																												■						
11282																																																		

Strain	Acquired β-lactamases																																			
	Carbapenemases										Extended Spectrum β-lactamases																		Penicillinases							C
	MBL									OXA	Class A										Class D								A	Class D						C
	IMP-1	IMP-13	IMP-26	VIM-1	VIM-2	VIM-4	AFM-5	NDM-1	DIM-1	OXA-641	SHV-2a	PER-1	PME-1	VEB-1	VEB-9	VEB-20	GES-1	GES-9	GES-27	ACA-7	OXA-17	OXA-19	OXA-35	OXA-142	OXA-520	OXA-677	OXA-1049	OXA-1170	CARB-2	OXA-2	OXA-4	OXA-9	OXA-10	LCR-1	NPS-1	PAC-1
9124																											■									
9556																																	■			■
9898																					■			■												
10893								■																									■			■
11558	■																																■			■
11596												■																								
SD974-21																																	■			■
NIC-15																																				
11504																		■																■		
CHA-08																																				
9104																																				
9246																																				
10088																												■								
10284																																				
10326																																				
PSA-19								■																												
10002								■																												
10340																																				
10391																																				
10615								■																												
10840								■																												
10992												■																								
11095								■																												
11191								■																												
11200																							■													
11232								■																												
11240								■																												
11260								■																												
11268								■																												
11273																																				
11286								■																										■		
11345								■																												
11359								■																												
11384								■																					■				■			
11402																																				
11462																■																				
11485			■				■													■																
11486								■					■																							
11531																																				
11533																						■														
11586								■																												
2963																						■														
8502								■																												
9023																																				
9148								■																												
9159						■		■																												
9460								■																												
9611						■				■																			■							
9731												■																			■					
9917								■																												
10070																						■							■							
10311									■						■																					
10650																																				
10845																																				
10900								■																												
10984								■							■																		■			
11016				■																										■						
11052								■										■																		
11119																																				
11173								■																												
11282																			■																	

11334	Bone			1047											■																																																																			■																																	■			
11393	Respiratory			773										■																																																																								■																																
11433	Other			357					■						■																																																																																			■																					
COR-15	Other			4156	■		■																																																																																																															
LIE-07	Respiratory		■	981									■		■															■																																																																																						
LYO-09	Respiratory		■	179					■				■										■																																																																					■																			■					
SD93-04	Urine			357	■		■		■						■																																																																																																		■		
6598	Screening		■	233											■																																																																																						■														
8610	Blood		■	111																																																																												■																												■	■					
8014	Blood			235			■																																																					■																																							■														
8643	Blood		■	272						■																																																																													■																						
8808	Respiratory			357					■						■																																																																													■																					
8928	Respiratory			207									■																																												■																																																												
9063	Respiratory			357					■		■				■																																																																												■																					
9321	Respiratory		■	111																																																																												■																												■	■					
9350	Skin			235																																																																																■											■																■			
9484	Respiratory			357					■						■																																																																											■																					
9579	Blood			357	■		■		■						■																																																																																																		■		
9769	Bone		■	179									■										■																																																																			■																			■					
10061	Blood		■	252	■	■							■																																																																																																											
10355	Respiratory			773																																																																																			■																																
10385	Skin		***	357					■						■																																																																																	■																		■	
10438	Skin			3559	■	■			■																																																								■																																																									
10539	Screening			357					■						■																																																																												■										■										
10562	Blood	4		534			■																																										■							■																											■																								
10630	Respiratory			560	■	■				■	■																							■								■		■																																																																							
10953	Blood			534			■																																										■																																					■																								
10976	Blood			235			■																																																																																			■																									
11060	Urine			773																																																																																				■																																
11087	Respiratory			235																																																																																												■																			
11164	Skin		■	111																																																																																																		■						■			■				
11165	Respiratory		■	4909	■				■		■													■																	■															■											■				■																																																		
11203	Urine			357					■						■																																																																		■									■									■											
11218	Blood			298																																																										■																							■																								
11221	Bone		■	412										■																			■						■																																		■																																														
11315	Blood			773								■			■																																																																				■				■																								
11371	Urine		■	179									■										■																								■																																													■																								
11512	Respiratory		■	4934	■					■		■				■																									■																																																																																	
11549	Urine			235																																																																																												■																			
11595	Other		■	233																																																																																		■							■																			■					
BET-15	Respiratory			253																																																																																																															
AMI-01	Respiratory		■	27	■		■		■			■			■																																																																																																							

* The strain lacked both *exoS* and *exoU* genes, harbored the *exlA* gene; and its genetic background corresponded to the C3 population, similar to the strain PA7

**PiuA and PiuD are orthologous proteins

*** The strain lacked both *exoS* and *exoU* genes

TABLE S3 Cefiderocol susceptibility in *Pseudomonas aeruginosa* PAO1 and deletion mutants of siderophore receptors and their regulators.

Strain and mutant	MIC of cefiderocol (mg/L)
PAO1	0.125
PAO1Δ*pirR*	0.5
PAO1Δ*piuC*	2
PAO1Δ*fptA*	0.25
PAO1Δ*pfeA*	0.125
PAO1Δ*fpvA*	0.06
